# King Edward's Hospital Fund for London

**Published:** 1904-11-26

**Authors:** Hugh C. Smith

**Affiliations:** Chairman of the Executive Committee


### KING EDWARD'S HOSPITAL FUND FOR LONDON.

### NEEDS FOR 1904.

*A sum to yield £14,000 per annum required to make up a permanent fixed income of £50,000 a year.*

We have received the following letter from Mr. Hugh C. Smith, chairman of the Executive Committee, which we refer to in another column :—

Before it is too late may I be allowed once more to call the attention of the charitable public to the munificent offer which has been made to this Fund, and which, if they enable us to avail ourselves of it, will be of great and lasting value to the sick poor of London.

As announced by his Royal Highness the Prince of Wales on March 8th last, an anonymous donor has offered to place at the disposal of this Fund an amount which will produce an income of £4,600 per annum, on condition that by December 31st next a further sum which would produce an income of £9,400 is collected. The object of the gift is to raise the permanent income of this Fund to £50,000 a year, with a view to enabling us ultimately to make an annual distribution of £150,000 a year.

The importance of this offer to the public is obvious. We were enabled last year to distribute £100,000, but only by drawing £18,750 from our capital. We have not felt justified in distributing the same amount this year, and consequently only £80,000 will be distributed. This is totally inadequate for the pressing needs of the hospitals.

His Royal Highness the President earnestly trusts that this opportunity of placing the Fund in such a favourable position will not be lost through lack of adequate response from the public.

We shall be glad to give further information to any who desire it, and ask that all communications may be addressed either to myself or to the honorary secretaries of King Edward's Hospital Fund for London, 81 Cheapside, E.C.

I am, Sir, your obedient servant,

Hugh C. Smith,
Chairman of the Executive Committee.

